# Abscesses of the Vulva Cured by Sepia

**Published:** 1892-12

**Authors:** I. Dever

**Affiliations:** Clinton, N. Y.


### ABSCESSES OF THE VULVA CURED BY SEPIA.

I. DEVER, M. D., CLINTON, N. Y.

November 30th, 1891, Mr. R—— came to me with a request that I call to see his wife, who had just returned from a fever-infected district in Northwestern Pennsylvania. He stated that his wife had been sick with a slow fever for ten weeks, and notwithstanding the fact that she had been constantly attended during the whole time by an able physician, he failed to bring about a salutary result, and had advised her to go North and escape the disease-producing influence of the atmosphere in which the fever had been contracted as the only hope of her recovery.

The husband was not long in giving me to understand that neither himself nor wife had any faith in Homœopathy, but his mother had advised him to call me, and he supposed that I would not confine myself to Homœopathy in so desperate a case.

He was an intelligent gentleman and I gave him a short lecture on Homœopathy, "pure and simple," which I practically demonstrated by curing his wife of her fever in three weeks. But for seven years she had been the subject of abscesses of the vulva, for the cure of which she had suffered much from the hands of many physicians, who had in no case withheld the use of the knife, but had cut into the abscesses both before and after the formation of pus, and by this and other harsh measures had greatly increased her sufferings, yet had not benefited her in the least.

The history of this case, like all others which have their origin in psora, dated back to childhood, and the footprints of psora could even be traced to her parents, who transmitted to her this undesirable inheritance.

She stated that when a child she suffered from an eruption of itching pimples, which had for their seat the inside of the joints, especially the bends of the elbows and knee joints. After puberty she was less troubled with the itching eruption except at the time of her menstrual periods it would sometimes, though not always, make its appearance on the hands and wrist.

Her menstrual periods were not regular, being one or two days late and always preceded by pain, always relieved by the flow. She complained of a bearing-down pain with stitching or shooting pains from below upwards toward the umbilicus and pit of the stomach.

During her inter-menstrual period she was troubled with an itching, smarting, and burning leucorrhœa. Her urine became turbid and very offensive after standing, and deposited an adhesive *red* sediment on the side of the chamber.

She is twenty-eight years old and has been married four years. Has had abscesses of the vulva for seven years, but previous to her marriage she would not have them often—not more than one a year—but when she first consulted me she stated that she was at no time free from them, and as fast as one began to get well another appeared to torment her and make her life hardly worth living.

I did not examine the abscesses, but she described them as seated in the vulva, and on first appearance as being hard and extremely sensitive to the touch, and as large as a walnut. They were slow in pointing, but when they came to a head they discharged large quantities of a dirty brown or mahogany-colored pus.

She had spent some time in an allopathic hospital, where she received the full benefit of cuttings and hypodermic injections of caustics into the newly-formed abscesses, in the hope of aborting them, as her surgeon told her, but all to no effect, except to aggravate the difficulty and leave the lady hopeless and discouraged of ever obtaining relief, much less a cure. Such is the history as given by the lady, who freely confessed that she had about resigned herself to her condition, though she might possibly do as some of her friends had advised her, go and place herself under the care of some specialist and be knifed by more skillful hands.

After she had given me the above history of her case, I told her that the Homœopathy taught by Hahnemann and prescribed by his disciples—and there is no other Homœopathy—would cure her in a year, and if she wished, I would prescribe, providing she would faithfully follow my directions, to which she freely gave her hearty consent, and I began the study of her case, to follow it where the symptoms, as above narrated, would leave me. After an examination of the different remedies, I finally rounded up on Sepia as the simillimum for this particular group of symptoms, and prescribed Sepia$^{200}$, one dose, which I allowed to continue its action for six weeks. How well my prescription was sighted to the mark may be inferred from the following note received six weeks after my first prescription: "Doctor Dever, I have only had one abscess since I began your treatment. I am out of medicine and would be pleased if you would send me more. I am doing my own work. I feel well. Myself and husband are converts to Homœopathy." I continued the action of the Sepia by sending another dose 200.

March 3d, I received the following: "Please send me more medicine, I am getting along nicely, without abscesses, and I want the good work to go on."

My predecessors, the allopathic surgeons, did the best they knew—they treated the abscesses, the result of psora, with the knife, and failed. I treated the cause of the abscesses, the psora, with the homœopathic remedy—or in other words, the simillimum Sepia, and cured.

### Discussion.

Dr. Kennedy—Why did Dr. Dever repeat the remedy, when the patient was still improving?

Dr. Dever—The lady lived three hundred miles from me, and I sent the medicine in the way that I have described on account of my inability to see her often. I should have done differently, no doubt, under different circumstances.

Dr. Tompkins—I hope Dr. Kennedy does not find fault with the time in which that case was cured?

